# Phylogeny and Taxonomy of Allium Section *Longibidentata* (R.M.Fritsch) R.M.Fritsch (*A.* Subgenus *Melanocrommyum*): Resurrection of *Allium simile* Regel

**DOI:** 10.3390/plants15091289

**Published:** 2026-04-22

**Authors:** Nikolai Friesen, Daulet Sh. Abdildanov, Laura Shadmanova, Polina V. Vesselova, Nadezhda G. Gemejiyeva, Gulmira M. Kudabayeva, Ramina Akhmetzhanova, Akerke H. Kenesbay, Vladimir Epiktetov, Reinhard M. Fritsch

**Affiliations:** 1 Botanical Garden, University of Osnabruck, Albrechtstrasse 29, 49076 Osnabruck, Germany; 2 Institute of Botany and Phytointroduction, 36D Timiryazev Str., Almaty 050040, Kazakhstan; laura_shadmanova@mail.ru (L.S.); pol_ves@mail.ru (P.V.V.); ngemed58@mail.ru (N.G.G.); kgm_anita@mail.ru (G.M.K.); ramina.akhmetzhanova@bk.ru (R.A.); akerkekenesbay2026@gmail.com (A.H.K.); v.epiktetov@gmail.com (V.E.); 3 Faculty of Biology and Biotechnology, Al-Farabi Kazakh National University, Almaty 050040, Kazakhstan; 4 Leibniz Institut für Pflanzengenetik und Kulturpflanzenforschung (IPK), Corrensstrasse 3, 06466 Seeland OT Gatersleben, Germany; ritschrmgat@gmail.com

**Keywords:** *Allium fetisowii*, *A. simile*, section *Longibidentata*, phylogeny, Kyrgyz Alatau, Tian Shan, Central Asia

## Abstract

During fieldwork in the western Tian Shan Mountain range, somewhat different forms of *Allium fetisowii* s.l. were observed in its eastern and western parts. A detailed morphological study using principal component analysis (PCA) revealed the presence of two well-separated taxa within *A. fetisowii* s.l. A molecular study based on nrITS and four plastid markers (trnL–trnF, rpl32–trnL, trnQ–rps16 spacers, and the rps16 intron) confirmed their status at the species level. *Allium fetisowii* Regel s. str. occurs in the eastern part, whereas the name *A. simile* Regel applies to the plants growing in the western part. Together with *A. chychkanense*, these species constitute section *Longibidentata*, which is supported by molecular data. The nomenclatural history of these three species is explained. A taxonomic conspectus is provided, the distribution is mapped, and an identification key is presented.

## 1. Introduction

In early summer, the steppe slopes of the Tianshan mountain range display a colorful appearance dominated by tall forbs. Smaller plants may also create striking visual effects when they dominate certain patches, including several species of *Allium*, mostly from subgenus *Melanocrommyum*. During a detailed study of such *Allium* species in the Kyrgyz Alatau, it became apparent that *Allium fetisowii* Regel [1] differs slightly from plants bearing the same name that grow near Almaty in the Trans-Ili Alatau.

Initial sequencing of the ITS molecular marker in several plants from the Kyrgyz Alatau range, followed by comparison with published GenBank sequences, revealed two distinct clades: one consisting of plants from the Almaty region, and the other comprising plants from the Kurdai Pass, the Kyrgyz Alatau, and the western Tien Shan. This second clade is split into two sister clades: one containing *Allium chychkanense* R.M.Fritsch, recently described by R.M. Fritsch [2], and the other containing specimens from the Kyrgyz Alatau and additional material from Kyrgyzstan, the region from which *Allium simile* Regel [3] was originally described. These preliminary findings prompted us to examine more closely the nomenclature and relationships among these taxa.

In the protologue of *Allium simile*, Regel cited three herbarium specimens: “In valle fluvii Tschirtschik 5–7000′ alt. montium alatavicorum taschkendicorum (A. Regel, 21 June 1881); in montium alexandricorum angustiis Alamedin et Arassan, June 1880, 7–9000′ alt. (Fetisow); prope Wernoje (Krasnow).” The second specimen, collected in Kyrgyzstan, was designated as the lectotype by Reinhard Fritsch [4]. He noted: “Specimens from all three localities, identified in E. Regel’s handwriting, are still present at LE. The specimen from Wernoe without doubt belongs to *A. fetisowii* Regel, and the one from the Tchirchik valley very likely belongs to *A. sewerzowii* Regel. From the Alexander Mountains, two sheets are present containing three collections: from the Alameddin Gorge, 23–25. V. 1880; from Mt. Arassan, 7–9000′, collected on 7 and 8. VI. 1880 (the latter being an isolectotype); and the above-cited lectotype.”

These specimens represent plants that occasionally show a third or fourth small tooth-like appendage at the lower part of the broadened base of the inner filaments. Eduard Regel was aware of this, as indicated on the specimen label, and considered the difference unimportant—thus supporting the lectotypification of *A. simile* by this specimen.

*Allium simile* was later treated as a synonym of *Allium fetisowii* by Vvedensky, who added the following commentary ([5]: 264, translated from Russian): “I was able to compare living specimens of *Allium fetissovii* from Ugam (*A. simile*) and from the Alma-Ata region (*A. fetissovii* s. s.). The Almaty specimen has slightly smaller flowers, significantly narrower tepals of a different shade (pink with a distinctly purple base, whereas the Ugam specimen has pinkish-violet tepals), a more rounded ovary, and darker filaments. Overall, the Ugam specimens are coarser and larger in all parts, but I had too little material to judge whether these differences are consistent.”

Later, Kamelin [6], in his florogenetic analysis of the mountain flora of Central Asia, recognized both species. Fritsch subsequently placed *Allium fetisowii* and the newly described *A. chychkanense* into a new section, *Longibidentata* (R.M.Fritsch) R.M.Fritsch [2], while retaining *A. simile* as a synonym of *A. fetisowii*. After studying living plants near Chirchik, he concluded that the syntype from this area certainly belongs to *A. tschimganicum* O.Fedtsch., not to *A. sewerzowii* [7].

The aim of this study is to clarify the nomenclature, taxonomy, and relationships within section *Longibidentata*. To achieve this, we conducted a morphological examination of all available herbarium specimens in the herbaria AA, GAT, and LE, and sequenced nrITS and four plastid DNA regions (the *trnL–trnF* and *rpl32–trnL* spacers, the *trnQ–rps16* spacer, and the *rps16* intron) from 12 accessions for phylogenetic analysis.

## 2. Results

### 2.1. Morphology and Distribution

Morphological measurements from herbarium material and our own collections of *A. fetisowii* and *A. simile* are presented in Table 1.

**Figure 1:**
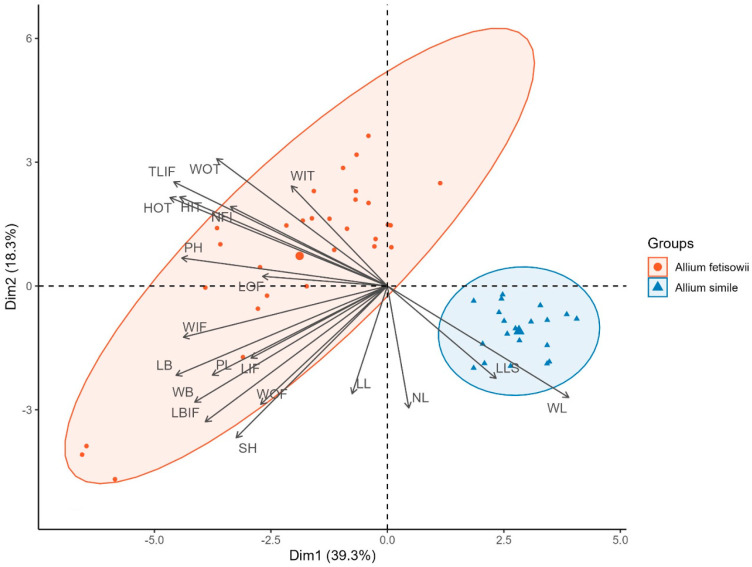
Principal component analysis (PCA) biplot illustrating morphological differentiation between *Allium fetisowii* and *Allium simile.* LOF*—*Length OF, LIF—Length IF, WOF—Width OF, WIF—Width IF, LB IF—Length of basal fusion of IF, PL—Pistil length, WIT—Width IT, WOT—Width OT, HIT—Height IT, HOT—Height OT, TLIF—Teeth length on the base of IF, PH—Pedicel length, SH—Scape height, NL—Number of leaves, WL—Width of leaves, LL—Length of leaves, LLS—Supraterranean length of leaf sheaths, WB—Width of bulb, LB—Length of bulb, NFI—number of flowers in the inflorescence.

The principal component analysis (PCA) based on the morphological measurements (Table 1) has clearly shown two morphotypes within *A. fetisowii* s.l. (Figure 1).

The two species were distinctly separated along the first component axis. *Allium simile* formed a compact, well-delimited cluster on the right side. This tight grouping reflects the morphological homogeneity of the species. The characters most strongly associated with *A. simile* were leaf parameters—leaf width (WL), leaf length (LL), number of leaves (NL), and supraterranean length of leaf sheaths (LLS)—whose vectors pointed clearly to the right and downward. This indicates that *A. simile* is characterized by a well-developed and relatively broad leaf apparatus.

*Allium fetisowii*, by contrast, occupied a broad area in the left part of the ordination diagram, forming an elongated ellipse oriented from the upper-left to the lower-left quadrant. The considerably greater scatter of points compared to *A. simile* reflects substantial intraspecific morphological variability. Several specimens were positioned well outside the 95% confidence ellipse, suggesting the possible presence of morphologically divergent forms or pronounced interpopulation variation within the species.

The character vectors associated with *A. fetisowii* fell into two distinct directional groups. In the upper-left sector, vectors corresponding to floral and perianth characters were concentrated—width of outer tepal (WOT), teeth length on the base of the inner filament (TLIF), height of outer tepal (HOT), height of inner tepal (HIT), number of flowers in the inflorescence (NFI), width of inner tepal (WIT), and pistil length (PL)—reflecting the importance of generative structures in characterizing this species. In the lower-left sector, vectors associated with bulb and filament parameters were grouped—width of bulb (WB), length of bulb (LB), length of basal fusion of inner filament (LBIF), width of inner filament (WIF), length of inner filament (LIF), width of outer filament (WOF), length of outer filament (LOF), and scape height (SH)—indicating that underground vegetative and reproductive structures also contribute substantially to the morphological identity of *A. fetisowii*.

The PCA biplot demonstrates that *A. fetisowii* and *A. simile* are differentiated by qualitatively distinct sets of morphological characters: leaf parameters are diagnostic for *A. simile*, whereas a combination of floral, perianth, bulb, and filament characters defines *A. fetisowii*. The minimal overlap of the 95% confidence ellipses confirms clear interspecific morphological differentiation and supports the taxonomic independence of both species.

**Figure 2:**
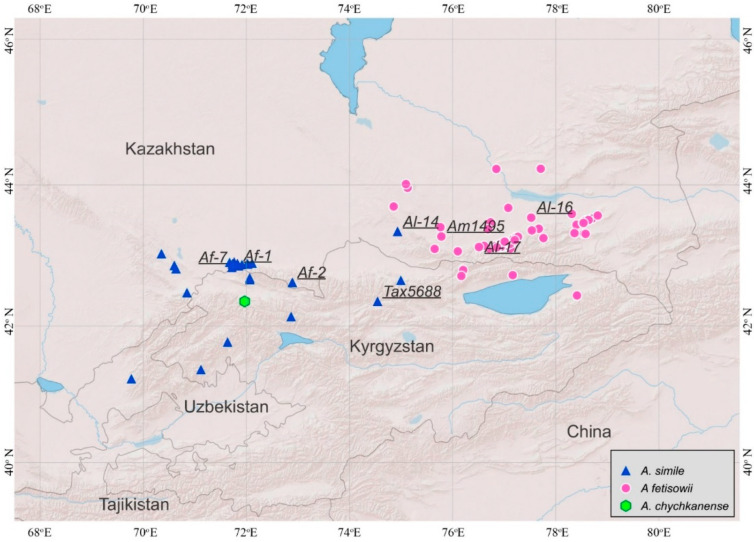
Distribution of the *Allium* species of section *Longibidentata.* The numbered points indicate the molecularly investigated accessions.

Analysis of the collection points in the herbarium collections (LE, AA and GAT) has revealed the distribution of the three species of the section *Longibidentata* (Figure 2, Table 2). A list of the examined herbarium specimens of species of the section *Longibidentata* is presented in Supplementary S1.

**Figure 3:**
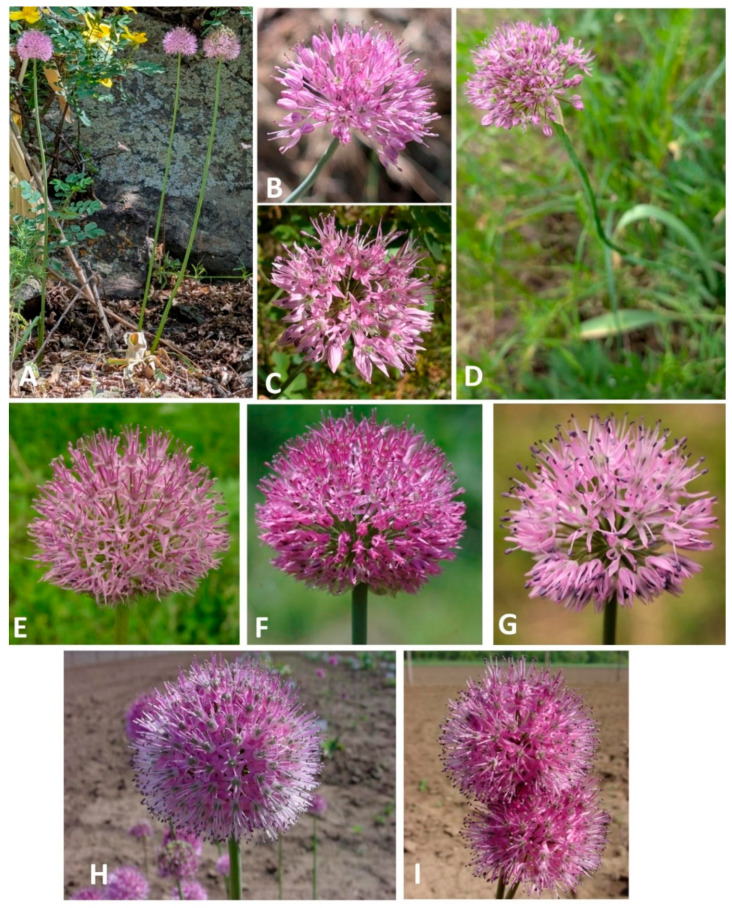
Species of *Allium* section *Longibidentata*: (**A**–**D**) *Allium simile*; (**E**–**G**) *A. fetisowii*; (**H**,**I**) *A. chychkanense*. (**A**,**C**,**D**) Fotos N. Friesen, (**E**–**G**) Foto V. Epiktetov, (**H**,**I**) Fotos R.M. Fritsch.

**Figure 4:**
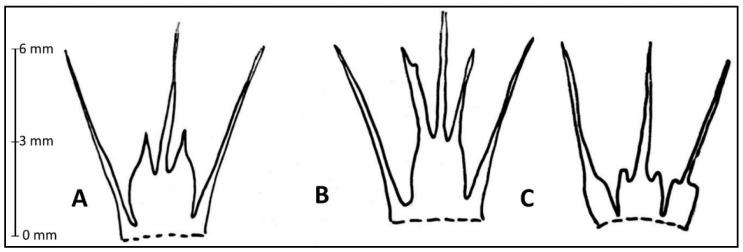
Filaments of *Allium* section *Longibidentata*. (**A**)—*A. simile*, (**B**)—*A. fetisowii*, (**C**)—*A. chychkanense*.

The shape, length, and width of the teeth in the inner filaments have proven to be stable morphological characteristics for distinguishing all three species currently recognized in the section *Longibidentata*. See Figure 3 and Figure 4 and some photos in Supplementary S1.

### 2.2. Phylogenetic Analysis

**Figure 5:**
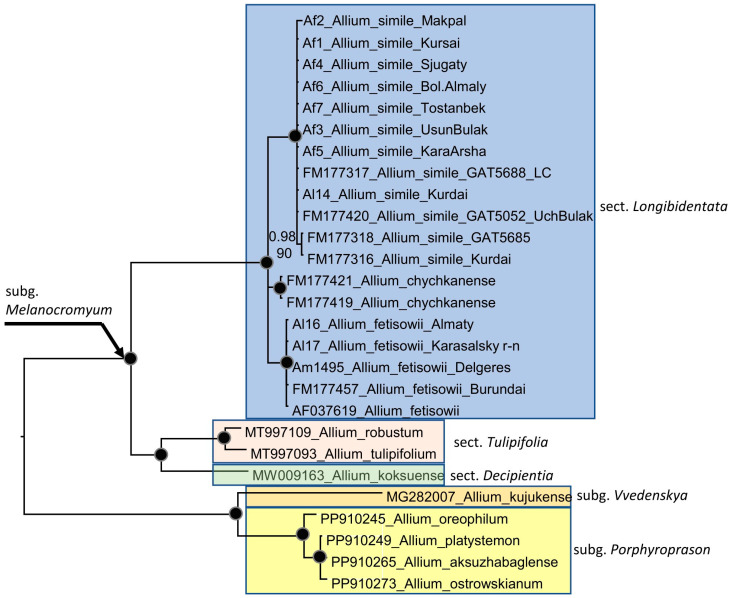
Phylogenetic tree based on ITS sequences of *Allium* subgenus *Melanocrommyum* section *Longibidentata*, including some related sections and the subgenera *Vvedenskya* and *Porphyroprason* as outgroups. Numbers at nodes represent Bayesian posterior probabilities (PP) and bootstrap support (BS, 1000 replicates). The joint presence of PP > 0.98 and BS > 95% is indicated with a black dot. For the origin of the Am and Tax sequences, see Table 2.

The alignments of ITS sequences (including the 5.8S gene) with 27 accessions (12 accessions are new sequences, Table 1) (Figure 5) consist of 642 characters, of which 223 variable characters are parsimony informative. Unweighted parsimony analysis of the 27 sequences resulted in 9 most parsimonious trees of 231 steps (CI = 0.889571). The substitution model TVM + G was chosen by AIC in jmodeltest-2.1.7 for the Bayesian analysis. Parsimony and Bayesian analyses produce identical topology.

All accessions of section *Longibidentata* formed three well-supported clades (each with 100% bootstrap support (BS) and posterior probability (PP) of 1), although the relationships among these clades were not resolved. One clade comprised plants from the Almaty region (*Allium fetisowii* s. str.). A second clade contained *A. chychkanense*, and a third clade included *A. simile* plants from the Kurdai Pass, the Kyrgyz Alatau, and the western Tian Shan, including the accession from the type locality of *A. simile*.

The clade containing species of sections *Tulipifolia* and *Decipientia* was recovered as the sister group to section *Longibidentata*. Phylogenetic analysis of individual plastid fragments did not yield as clear results as those obtained in the nrITS analysis (see Supplementary S2). In the *trn*L-*trn*F tree, all three species, *A. simile*, *A. fetisowii*, and *A. chychkanense*, form their own clades with relatively weak support. The clade containing *A. chychkanense* forms a sister clade with *A. fetisowii*, and the clade containing *A. simile* is separated from the clades of *A. fetisowii* and *A. chychkanense* by species from the sections *Decipientia* and *Tulipifolia*, including the accession of *A. simile* from the type location.

Because we were unable to sequence other plastid fragments for *Allium chychkanense*, accessions of this species are missing from further individual analyses and from the combined plastid tree.

The phylogenetic trees based on the *rpl*32-*trn*L^(UAG)^ and *trn*Q-*rps*16 spacers are almost identical, with a slight difference; the accessions of *A. fetisowii* in the *trn*Q-*rps*16 tree do not form a clade. The very strongly supported clade with *A. simile* accessions forms a sister clade with *A. fetisowii* accessions, and together they form a sister clade to species from the sections *Decipientia* and *Tulipifolia*. The sequences of the plastid rps16 intron showed significant differences among the accessions of *A. simile*. The accession Af3 from Uzun-Bulak in the western part of the Kyrgyz Alatau forms a sister clade to a very strongly supported *A. fetisowii* clade, and together they form a sister clade to a much weaker supported *A. simile* clade.

Within the *A. simile* clade, there are at least three subclades: Accessions Af6 and Af7 form a sister clade to all other *A. simile* accessions. In this main clade, the accessions from Kurdai Pass Al4 and Kursai valley Af1, together with Tax5688 from the type location, form a relatively strongly supported subclade (PP 0.91 and BS 88).

**Figure 6:**
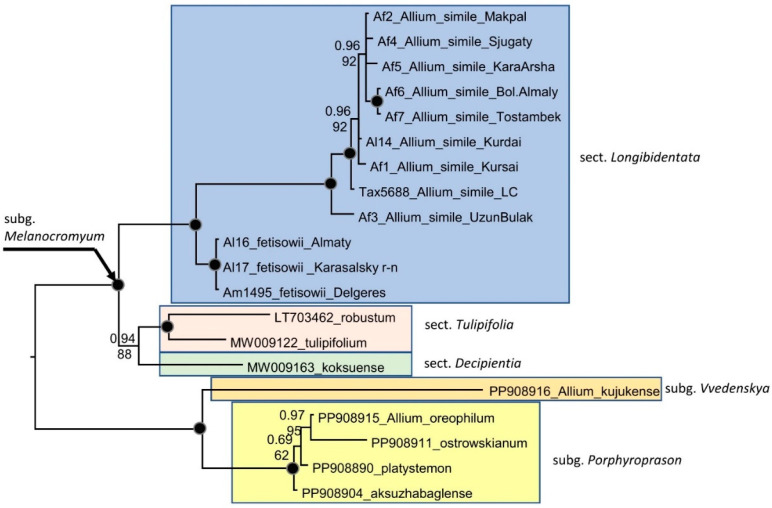
Phylogenetic tree of section *Longibidentata*, including some related sections and the subgenera *Vvedenskya* and *Porphyroprason* as outgroups based on combined chloroplast fragments (*rpl32-trnL*^(*UAG*)^, *trnQ-rps16*, *trn*L-*trn*F spacers and *rps16 intron*). Numbers at nodes represent Bayesian posterior probabilities (PP) and bootstrap support (BS, 1000 replicates). The joint presence of PP > 0.98 and BS > 95% is indicated with a black dot. For the origin of the Af, Al, Am and Tax sequences, see Table 2.

The alignment of combined plastid sequences of *trn*L-*trn*F, *trn*Q-*rps*16, *trn*L-*rpl*32 spacers and *rps*16 intron based on 20 accessions consists of 3432 characters (Figure 6). Unweighted parsimony analysis of the 20 sequences resulted in 2 most parsimonious trees of 157 steps (CI = 0.780000). The GTR+G substitution model was selected by AIC in jmodeltest-2.1.7 for the Bayesian analysis. Both analyses, Parsimony and Bayesian, produced identical topology. All accessions of section *Longibidentata* revealed two distinct clades with strong support: one clade included plants from the Almaty region (*A. fetisowii* s. str.), and the other included plants from the Kurdai Pass, the Kyrgyz Alatau, and the western Tian-Shan (*A. simile*).

## 3. Discussion

Morphological and phylogenetic analyses clearly show that *Allium simile* is a distinct species and should be accepted. Thus, section *Longibidentata* consists of three species: *Allium fetisowii*, *A. simile* and *A. chychkanense*, and is next related to the sections *Tulipifolia* and *Decipientia* [8]. While the nuclear DNA analysis (nrITS) shows hardly any difference between *A. simile* accessions, and all three species of section *Longibidentata* are strongly monophyletic (Figure 4), the plastid analysis also shows all three species to be clearly monophyletic, but accessions of *A. simile* show more variability (Figure 5) what could indicate hybridization events in these accessions. The fact that different chloroplast fragments can show very different phylogenetic signals is not new in *Allium*. In the largest phylogenetic analysis of the subgenus *Amerallium* [9], the four plastid fragments (*trnL-trnF*, *atpB-rbcL*, *rpl32-trnL*, *rps16* intron) showed different topologies in the plastid trees. Only in the combined analysis was the plastid tree comparable to the nuclear ITS tree.

It should be mentioned here that the distribution boundary between *A. fetisowii* and *A. simile* fits very closely to the boundary between two phytogeographical regions, namely the Western Tian Shan and the Northern Tian Shan, which was recently determined [10], which confirms that these two species have distinct evolutionary histories. The limit between the Western Tian Shan and the Northern Tian Shan cuts the Kyrgyz Range approximately at 73.5°. This separation of the West Tian Shan and Northern Tian Shan phytogeographical regions was determined according to the distribution boundaries of several taxa and also vegetation types in the Tian Shan. Our results provide further evidence that both species have their own distinct phylogenetic history.

### 3.1. Taxonomy

*Allium* sect. *Longibidentata* (R.M.Fritsch) R.M.Fritsch, Bot. Jahrb. Syst. 127(4): 465 (2009) [2].

Type: *Allium fetisowii* Regel

***Allium fetisowii*** Regel, Trudy Imp. S.-Peterburgsk. Bot. Sada 5: 631 (1878) [1].

Lectotype: Ex horto bot. Petropolitano 78.5 “Von Fetisow Zwiebeln aus Wernoje erhalten” [Fetisow sent the bulbs from Vernoye] (LE, design. Fritsch 1990: 504).

Distribution: Kazakhstan, Kyrgyzstan, China: W Xinjiang: N Tianshan mountain ranges: from 75 degrees east longitude to the Karasu River in the East, colline to submontane grassy and steppe slopes, with not too dense shrubs and open places.

***Allium simile*** Regel, Trudy Imp. S.-Peterburgsk. Bot. Sada 10: 359 (1887) [3].

Lectotype: Kyrgyzstan: Angust. Arassan. m. Alexander [Arassan gorge of Alexander Mts.], 7–9000′, 7.6.1880, leg. Fetisow (LE, design. Fritsch 1990: 507).

Distribution: Kazakhstan, Kyrgyzstan and Uzbekistan. From Kyrgyz Alatau and Kurdai Pass in the East to the western part of Kyrgyz Alatau and West Tianshan, colline to submontane grassy and steppe slopes, with not too dense shrubs and open areas.

***Allium chychkanense*** R.M.Fritsch, Bot. Jahrb. Syst. 127(4): 466–467 (Figure 3) (2009) [2].

Type: Cultivated in Gatersleben no. TAX5057, leg. 05.05.1999, plants collected in Kyrgyzstan, Talassischer Alatau, rechter Hang am Chychkan-Fluss ca. 15 km unterhalb des Alabel-Passes, trockene Stellen am Hang, ca. 2200 m, 42°15′ N, 73°00′ E, 03.07.1994 R.M. Fritsch, K. Pistrick & F.O. Khassanov no. 1206 (holotype GAT, isotypes GAT, TASH).

Distribution: Kyrgyzstan: Central Tian-Shan mountain ranges, moderately dry montane slopes and river terraces in higher altitudes of Susamyr massif, known only from the type area.

### 3.2. Key to Determine Allium Species in Sect. Longibidentata

Tepals shining pink, delicate, with a barely noticeable vein, 5–7 mm long; bases of inner staminal filaments widened and toothed
**sect. *Longibidentata.***
**1.** Leaf blades 17–20 (–30) cm long, 0.5–1 (–1.5) cm wide; tepals semi-reflexed, apices incurved
**2**
1* Leaf blades 20–25 cm long, 1.5–5 cm wide; tepals completely reflexed; all filaments widened at base and often with small, rounded teeth; inner filaments always toothed, outer filaments often widened but without teeth
*Allium chychkanense*
**2.** Expanded bases of inner filaments with long, sharp teeth, teeth nearly as long as the width of the filament base
*Allium fetisowii*
2* Expanded bases of inner filaments with short, pointed teeth
*Allium simile*


## 4. Materials and Methods

### 4.1. Morphological and Distribution Analyses

Field studies were conducted in 2025 within the investigated territories. Distribution maps were compiled from analyses of herbarium collections, including the authors’ own field collections.

During the fieldwork, herbarium material and specimens of representatives of the genus *Allium* L. occurring in the study area were collected. In total, 20 individuals of *Allium simile* (from 10 herbarium sheets) and 30 individuals of *Allium fetisowii* were morphologically analyzed (see herbarium sheet data in Supplementary S3). Plant measurements were taken with a ruler and a hand lens; mean values, standard deviations and standard deviation were calculated in Microsoft Excel using the Data Analysis tool.

Identification of the collected material was performed based on fundamental taxonomic works on the genus *Allium*, taking into account recent publications on species recorded in the Kyrgyz Alatau [11,12,13]. Herbarium collections of the Institute of Botany and Phytointroduction (AA, Almaty, Kazakhstan), Lomonosov Moscow State University (MW, Moscow, Russia), and the Komarov Botanical Institute (LE, Saint Petersburg, Russia) were examined.

The databases International Plant Names Index [14] and Plants of the World Online [15] were also consulted.

To identify morphological differentiation between *A. fetisowii* and *A. simile*, Principal Component Analysis [16] was performed on a standardized matrix of 20 morphological characters. Prior to PCA, multicollinearity among morphological characters was assessed by examining pairwise correlations. No pair of characters exceeded the correlation threshold of r > 0.90, and therefore all characters were retained for the analysis. Prior to analysis, all variables were standardized to a zero mean and unit variance to account for differences in measurement units and scale. PCA was performed using the prcomp() function in R v4.4.1 [17]. Results were visualized as a biplot displaying the ordination of individual specimens and the factor loadings of the original characters in the space of the first two principal components simultaneously. Confidence ellipses were constructed at the 95% probability level for each taxonomic group.

Data mapping was carried out using QGIS software, version 3.34.13 (https://qgis.org; accessed on 10 February 2026).

### 4.2. DNA Extraction, PCR Amplification, and Sequencing

Total genomic DNA was isolated from herbarium specimens, and silica-dried leaves collected by the authors using the “InnuPREPP Plant DNA Kit” (Analytic Jena AG) (Jena, Germany) according to the manufacturer’s instructions, and was used directly in PCR amplifications. Herbarium vouchers of all used accessions for phylogenetic analysis were deposited in the herbarium of the Institute of Botany and Phytointroduction, Almaty (AA). The complete ITS region (ITS-1, 5.8S and ITS-2) was amplified using primers ITS-A [18] and ITS-4 [19]. The PCR conditions followed Friesen et al. [20]. PCR conditions for the plastid region and primers were as follows: for *trn*L-*trn*F spacer as described in Taberlet et al. [21], for the *rp*L32-*trn*L (UAG) and *trn*Q-*rps*16 spacers, as described in Shaw et al. [22], for *rps*16 intron as described in Oxelman et al. [23], for *trn*L-*trn*F spacer as described by Taberlet et al. [18]. PCR products were sequenced on the SeqStudio Genetic Analyser using a 4-capillary run (ThermoFisher, Scientific, Waltham, MA, USA https://www.thermofisher.com/de/de/home/life-science/sequencing/sanger-sequencing/genetic-analyzers/models/seqstudio.html) (accessed on 13 February 2026). Forward and reverse sequences from each individual were manually edited in Chromas Lite 2.1 (Technelysium Helensvale, Queensland, Australia) and combined into single consensus sequences. The sequences of all samples were aligned using Clustal X [24], and the alignment was subsequently manually corrected in Mega 7 [25]. GenBank accession numbers of new sequences are shown in Table 2.

### 4.3. Phylogenetic Analysis Methods

Phylogenetic analysis was carried out on both individual and combined datasets (nuclear ITS and plastid DNA sequences) using parsimony and Bayesian methods. The amplified ITS sequences of all species in the section *Longibidentata* were included in a taxonomically revised ITS dataset from GenBank, representing some species of the subgenus *Melanocrommyum* [8,26,27,28]. Accession numbers shown in Table 2. In the individual analysis of plastid fragments, only accessions from section *Longibidentata* and the related sections *Decipientia* (Omelczuk) R.M. Fritsch and *Tulipifolia* R.M. Fritsch & N. Friesen [8], besides outgroup taxa, were included. In the combined plastid analysis, only our own sequences from section *Longibidentata*, excluding *A. chychkanense*, were used. As outgroup taxa, we used species of *Allium* subgenera *Vvedenskya* (Kamelin) R.M. Fritsch and *Porphyroprason* (Ekberg) R.M. Fritsch [29]. Parsimony analysis was performed in PAUP* 4.0b10 [30] using heuristic searches with TBR (Tree Bisection-Reconnection) and 100 replicates of the random addition sequence. Bootstrap support [31] was estimated with 1000 bootstrap replicates. Bayesian analyses were implemented with MrBayes 3.1.23 [32,33]. Sequence evolution models were evaluated using the Akaike Information Criterion (AIC) with the aid of jModelTest2 v2.1.6 [34]. Two independent runs, each with eight chains, 10 million generations, and sampling every 1000 generations, were executed. 25% of the initial trees were discarded as burn-in. The remaining trees were combined into a 50% majority-rule consensus tree.

Both alignments (nrITS and combined plastid) can be seen in the Supplementary S4 and S5.

## 5. Conclusions

Field and molecular studies have confirmed that within the *Allium fetisowii* s. l. complex, two clearly distinct species can be recognized: the eastern *Allium fetisowii* s. str. and the western *Allium simile*. Morphological traits (including tepal size, pistil length, and filament structure), together with molecular evidence, reliably support their species-level distinction. Therefore, *Allium simile* should be treated as a separate, well-defined species within the sect. *Longibidentata* of subgenus *Melanocrommyum*. The distribution boundary between the two species also fits exactly with the boundary between the phytogeographical regions in Tian Shan, which is further evidence for the separate phylogenetic history of *A. simile* and *A. fetisowii*.

## Figures and Tables

**Table 1:** Comparison of morphological characters of *A. fetisowii* (30 individuals) and *A. simile* (20 individuals).

Characters	*Allium fetisowii*	*Allium simile*
Length OF (mm)	1.10 ± 0.16	0.95 ± 0.18
Length IF (mm)	1.17 ± 0.15	1.10 ± 0.08
Width OF (mm)	0.40 ± 0.15	0.33 ± 0.08
Width IF (mm)	0.55 ± 0.15	0.45 ± 0.13
Length of basal fusion of IF (mm)	0.39 ± 0.16	0.33 ± 0.10
Pistil length (mm)	2.40 ± 0.50	2.10 ± 0.08
Width IT (mm)	2.08 ± 0.26	2.05 ± 0.05
Width OT (mm)	2.08 ± 0.14	1.80 ± 0.39
Height IT (mm)	5.10 ± 0.30	4.80 ± 0.20
Height OT (mm)	6.10 ± 0.21	5.70 ± 0.05
Teeth length on the basis of IF (mm)	2.10 ± 0.4	1.50 ± 0.05
Pedicel length (cm)	1.59 ± 0.21	1.30 ± 0.40
Scape height (cm)	34 ± 9.50	33.1 ± 3.10
Number of leaves	(1–2) 3 1.14 ± 0.45	(1 or) 2 1.33 ± 0.51
Width of leaves (cm)	1.30 ± 0.20	1.5 ± 0.30
Length of leaves (cm)	17 ± 4.20	20 ± 2.10
Supraterranean length of leaf sheaths (cm)	0.30 ± 0.04	0.35 ± 0.06
Width of bulb (cm)	0.76 ± 0.43	0.46 ± 0.12
Length of bulb (cm)	1.27 ± 0.43	0.86 ± 0.23
number of flowers in the inflorescence	36 ± 9.60	26.6 ± 3.80

Note: Quantitative characters were measured in mm ± standard deviation; alternative data are given in brackets. OF—outer filament; IF—inner filament; IT—inner tepals, OT—outer tepals.

**Table 2:** Origin, source, and GenBank accession numbers used for phylogenetic analyses.

Accession	Species	Coordinates N, E	nrITS	*trn*Q-*rps*16	*rpl*32-*trn*L	*rps*16	*trn*L-*trn*F
Af-1	*A. simile*	42.8624315, 71.8461069	PX936607	PX971582	PX981434	PX961800	PX961811
Af-2	*A. simile*	42.623830, 72.890854	PX936608	PX971583	PX981435	PX961801	PX961812
Af-3	*A. simile*	42.9135228, 71.7615473	PX936609	PX971584	PX981436	PX961802	PX961813
Af-4	*A. simile*	42.8624315, 71.8461969	PX936610	PX971585	PX981437	PX961803	PX961814
Af-5	*A. simile*	42.890988, 71.824529	PX936611	PX971586	PX981438	PX961804	PX961815
Af-6	*A. simile*	42.8862973, 71.7286359	PX936612	PX971587	PX981439	PX961805	PX961816
Af-7	*A. simile*	42.8378927, 71.7171797	PX936613	PX971588	PX981440	PX961806	PX961817
Al-14	*A. simile*	43.3370598, 74.9284438	PX936614	PX998187	PX981441	PX961807	PX961818
Al-16	*A. fetisowii*	43.541389, 77.525024	PX936615	PX971590	PX981443	PX961808	PX961819
Al-17	*A. fetisowii*	43.127278, 76,512168	PX936616	PX971591	PX981444	PX961809	PX961820
Am1495	*A. fetisowii*	43.275617, 75.781708	PX936617	PX971592	PX981445	PX961810	PX961821
Tax5688	*A. simile* LC	42.35, 74.54	FM177317	PX971589	PX981442	LCPX967011	FN178009

## Data Availability

The original contributions presented in this study are included in the article/Supplementary Material. Further inquiries can be directed to the corresponding authors.

## Supplementary Materials

The following supporting information can be downloaded at: https://www.mdpi.com/article/10.3390/plants15091289/s1, Supplementary S1: Photos of flowers from herbarium specimens: A–C *Allium simile*; D–F *A. fetisowii.* Supplementary S2: Single CP Fragment trees. Numbers at nodes represent Bayesian posterior probabilities (PP) and bootstrap support (BS, 1000 replicates). The joint presence of PP > 0.98 and BS > 95% is indicated with a black dot. For the origin of the Af, Al, Am and Tax sequences, see Table 1. Supplementary S3: List of analyzed herbarium specimens of *Allium fetisowii*, *A. simile*, and *A. chychkanense* deposited in AA, MW, and LE. Supplementary S4. Fasta datei of nrITS Alignment of *Allium simile*, *A. fetisowii* and *A. chychkanense.* Supplementary S5. Fasta datei of combined alignment of trnL-trnF, rpl32-trnL, trnQ-rps16 spacers and rps16 intron of *Allium simile*, *A. fetisowii* and *A. chychkanense*.
