# Supplementary material for: Phylogeny and Taxonomy of Allium Section Longibidentata (R.M.Fritsch) R.M.Fritsch (A. Subgenus Melanocrommyum): Resurrection of Allium simile Regel
Source: Plants (Basel). 2026 Apr 22;15(9):1289. doi: 10.3390/plants15091289 (PMC13165040; doi:10.3390/plants15091289)
Supplement: Supplementary file 1 [file plants-15-01289-s001.zip › Supplementary S3.pdf]

Species	Label	N	E	Date of collection	Collectors	Herbarium
A. fetisowii	Semirechensk region, Verny	43.125034	76.927954	12.04.1902	Saposhnikov V.	LE
A. fetisowii	Trans-Ili Alatau, Almamtinki River valley	43.168175	76.988174	853	Saposhnikov V.	LE
A. fetisowii	Verny, foothills near the city	43.167547	76.939736	15.05.1906	Sokalsky N. D.	LE
A. fetisowii	Trans-Ili Alatau	43.200173	77.038797	15.05.1906	Sokalsky N. D.	LE
A. fetisowii	Verny, near the city	43.361884	77.537542	13.05.1908	Taranenko	LE
A. fetisowii	Semirechensk region, Verny	43.165994	76.927319	27.05.1908	Ptaschiki M.I.	LE
A. fetisowii	Almaty, near the Verny foothills	43.127442	76.937917	01.05.1909	Koltsov L. I.	LE
A. fetisowii	Verny, southern cities, chernozem soil, subsurface forest	43.167056	76.926211	11.05.1909	Bogolyubov S. N.	LE
A. fetisowii	Verny, near the city	43.121843	76.862801	22.05.1909	Bogolyubov S. N.	LE
A. fetisowii	Trans-Ili Alatau, Butakovsky gorge	43.172064	77.113758	02.06.1909	Mickelson A.	LE
A. fetisowii	Semirechye Zailiyskiy Alatau, grassy slopes	43.128338	76.935062	20.05.1912	Shishkin. B.	LE
A. fetisowii	Semirechye, Oi-tobolga gorge southern rocky slope	43.115572	76.860710	23.05.1912	Shishkin. B.	LE
A. fetisowii	Semirechye Kopalsk, right bank of the Ili River, Uch-Aral	44.222636	76.848536	29.05.1913	Shishkin B., Genina V.	LE
A. fetisowii	Semirechye, Kaskelena River gorge, grassy slopes	43.143761	76.611009	23.07.1913	Shishkin B., Genina V.	LE
A. fetisowii	Chu-Ili mountains, near the Besmoynak river, steppe	43.100236	75.651469	29.07.1914	V. Titova	LE
A. fetisowii	Towards the foothill steppe from the Verny Mountains	43.121991	76.859368	04.05.1916	Gorodetsky V. D.	LE
A. fetisowii	Surroundings of the Verny mountains, mountain black soil among shrubs	43.197270	77.033793	30.04.1928	Is unknown	LE
A. fetisowii	Trans-Ili Alatau, Almaty zapavednik, shaggy hill, steppe peak	43.224213	77.205776	30.06.1933	Popov M. G.	LE
A. fetisowii	Almaty region, Chilik district, Bala-Boguty mountains, southern slope 2 km west. Spring h-1200-1300m, rocky slope	43.593306	78.314246	04.06.1985	Grubov V. , Medvedeva N., Korobkov A., Tikhmenova I.	LE
A. fetisowii	Semirechensk region, Verny	43.202327	77.011014	23.05.1899	Killoman I.	LE
A. fetisowii	Kyrgyzstan, Susamyr district, Karakol River, lower terrace by the river. Across the Tipchak steppe	42.436467	78.413229	10.06.1951	Tkachenko V. I.	LE
A. fetisowii	Almaty neighborhood, sunny steppe slopes, south of the city	43.142046	76.835659	18.05.1928	Lipshitz S.	MW0814014
A. fetisowii	Almaty neighborhood, foothills of the Trans-Ili Alatau range	43.150207	77.040679	18.05.1928	Lipshitz S.	MW0814018
A. fetisowii	Almaty neighborhood, slopes on the right bank of the Almaty river	43.124210	76.920282	28.05.1928	Lipshits S. Yu.	MW0814010
A. fetisowii	Almaty's surroundings	43.117164	76.922603	28.05.1928	Pavlov N.V.	MW0814011
A. fetisowii	Steppe near Koyanda on the Taldykorgan road	44.225659	77.709915	08.06.1928	Lipshitz S.	MW0814019
A. fetisowii	Almaty neighborhood, Turgen along the road	43.119070	76.919479	07.05.1932	Lipshitz S.	MW0814013
A. fetisowii	Almaty region, meadow slope, malaya Almatinka, near urosch. Medeo	43.150090	77.050201	13.06.1936	Pavlov N.V.	MW0814017
A. fetisowii	Keminsky district, right waterfall gap	42.800004	76.205254	28.05.1986	Kuvaev V.	MW0814008
A. fetisowii	Keminsky district, right waterfall gap, downhill	42.715849	76.170074	28.05.1986	Kuvaev V.	MW0814009

A. fetisowii	Kyrgyzstan, south-western spurs of the Trans-Ili Alatau, Kichik-Kemin River basin, Sasykbulak River valley, above the confluence of the Chichekbulak River	42.727637	77.167194	05.06.1988	Klyuikov E. V.	MW0814005
A. fetisowii	Semirechensk region. Vernensky u. Okr. g. Vernogo. Counters near the Pensioner's dacha.	43.185892	76.992936	29.04.1917	Kharin S.	AA
A. fetisowii	Surroundings of Verny. Chernozem counters.	43.183509	76.993351	02.06.1918	Not known	AA
A. fetisowii	Semirech. region of the Verny district. Near Gulinsky's dacha.	43.164155	76.994702	02.06.1918	Kharin S.	AA
A. fetisowii	Butakovskaya gap, aspen forest.	43.156399	77.109178	24.06.1918	Titov V. S.	AA
A. fetisowii	Semirechensk region. Vernensky uyezd. Sugatinskaya gap.	43.441859	78.404969	25.05.1920	Kharin S.	AA
A. fetisowii	Atlmaty district. Slopes of counters near the mountains of Almaty.	43.181188	76.997961	02.05.1928	Pavlov N. V.	AA
A. fetisowii	Atlmaty district. Foothills of the Trans-Ili Alatau range, sunny steppe slopes.	43.526678	78.701975	18.05.1928	Lipshits S. Yu.	AA
A. fetisowii	Atlmaty district. Hillsides on the right bank of the Almaty River.	43.678501	77.079779	20.05.1928	Lipshits S. Yu.	AA
A. fetisowii	Surroundings of Alma Ata. In the shade of spruce, in the belt of pure spruce forest, in Artil.the gorge.	43.172251	77.110013	01.06.1930	Not known	AA
A. fetisowii	Chu-Ili Mountains. At the stanzas. Andrakai, in the valley by the rocks.	43.698294	74.856045	18.05.1934	Popov M. G.	AA
A. fetisowii	Alma-Ata region. St. Chemolgan. Chiyevy thickets.	43.106022	76.710793	28.05.1934	Geld A. I.	AA
A. fetisowii	Trans-Ili Alatau. Kastek district, Kastek river gorge.	43.066171	76.101162	30.06.1936	Linchevsky O. A.	AA
A. fetisowii	Trans-Ili Alatau. Sughata mountains. Syugatinsky gorge.	43.503711	78.585297	07.05.1937	Dmitrieva A. A.	AA
A. fetisowii	Trans-Ili Alatau. Sughata mountains.	43.500349	78.585735	19.05.1937	Popov M. G.	AA
A. fetisowii	Trans-Ili Alatau. Syugatinsky mountains. Rocky slopes.	43.494345	78.566606	28.05.1937	Gorbunova E. P.	AA
A. fetisowii	Trans-Ili Alatau. 5. Syugaty Gorge. Side slits.	43.466430	78.529851	05.06.1937	Goloskokov V. P.	AA
A. fetisowii	Trans-Ili Alatau. Syugaty gorge.	43.508355	78.638023	20.06.1937	Gorbunova E. P.	AA
A. fetisowii	On the northern meadow-steppe slope of the foothills, near Aksai.	43.476190	76.736372	29.05.1941	Polyakov P. P.	AA
A. fetisowii	Chu-Ili Mountains. Gorge of the Kara-Sulu River (a tributary of the Cherbokty River and the Chu River) near the village. Krasnogorsky district. On the southern fine-grained rocky slopes.	43.959447	75.125688	29.05.1942	Goloskokov V. P.	AA
A. fetisowii	Trans-Ili Alatau, Karakastek river. On the slopes of the mountain.	44.011411	75.095105	27.06.1951	Baitenov M. S.	AA
A. fetisowii	Trans-Ili Alatau. Central part of the southern slope of hr. Turaigyr, south-east of the height of 2132 m, along the	43.316470	78.546022	25.05.1953	Goloskokov V. P.	AA

	fine-grained beds of the side crevices to a height of 1500 m.					
A. fetisowii	Trans-Ili Alatau. Central part of the northern slope of hr. Turaigyr, near the pass (1946 m). On the steepened bottom of the flake.	43.312725	78.579344	01.06.1953	Goloskokov V. P.	AA
A. fetisowii	Eastern spurs of the Trans-Ili Alatau, Syugaty Mountains, a gorge near the cordon, along the steepened slopes of hills.	43.467371	78.540023	11.06.1955	Goloskokov V. P.	AA
A. fetisowii	Eastern spurs of the Trans-Ili Alatau. Southern slopes of hr. Big Boguts . On the site of a melted snowfield near the perevalnaya saddle.	43.571254	78.815518	16.06.1955	Goloskokov V. P.	AA
A. fetisowii	South-west.the end point. Trans-Ili Alatau. Middle course of the Karakunuz River. On the northern grassy slopes.	43.239780	77.178488	14.06.1963	Goloskokov V. P.	AA
A. fetisowii	Trans-Ili Alatau. Turgen gorge, near the Turgen hot spring.	43.253263	77.761987	16.05.1967	Tsagolova V. G.	AA
A. fetisowii	Trans-Ili Alatau, right Talgar, south-western slopes. Height 1800-1900 m.	43.269818	77.233516	06.06.1967	Tsagolova V. G.	AA
A. fetisowii	Trans-Ili Alatau, Kaskelen river gorge, grass-mixed slope.	43.379271	76.686201	19.06.1993	Adilbayeva	AA
A. fetisowii	Trans-Ili Alatau. Eastern outskirts of Talgar. Height 950. Heavily dissected loess foothills.	43.271416	77.268749	14.05.1994	Danilov M. P.	AA
A. fetisowii	Trans-Ili Alatau. Eastern edge of Karakemir village, 900 m high. Sagebrush on loess foothills.	43.385443	77.672973	25.05.1994	Danilov M. P.	AA
A. fetisowii	Almaty region, Trans-Ili Alatau, Kokpek pass h~810m.	43.32295	78.36390	03.05.2018	Edina I. A., Otradnykh I. G., Bilibayeva B. K., Akhatayeva D. A.	AA
A. fetisowii	Almaty region, Zhambyl district, outside the village. Targap, before turning to Kopa, h~769m	43.406657	75.765104	18.05.2018	Edina I. A., Otradnykh I. G., Bilibayeva B. K., Akhataeva D. A.	AA
A. fetisowii	Almaty, Zailiysky Alatau, SSPE "Medeu", height 1387m	43.107670	77.12050	18.05.2020	Danilov M. P., Vesselova P. V., Kudabayeva G. M., Osmonali B. B.	AA
A. simile	Talas Alatau, Ulken Kaindy gorge, 2900 m.	42.477950	70.845220	06.07.1933	Linchevsky I. A.	LE
A. simile	Kyrgyz Alatau, Taldysu, bolshetravye	42.886133	72.024631	17.06.1931	Massagetov, Massalsky district	LE
A. simile	Kyrgyz Alatau, Mamai-Kaindy, subalpine zone, meadow area	42.890503	72.008626	15.06.1930	Yul'nin G. I.	LE
A. simile	Kyrgyz Alatau, Sulutor gorge at an altitude of about 1000 m.	42.898037	72.109812	31.04.1930	Zapryagaev F. L.	LE
A. simile	Alexander ridge, Taldybulak, alpine and subalpine zones	42.877521	71.911989	12.07.1924	Popov M. G.	MW0814016
A. simile	Karatau mountain range, Aktas, surrounded by shrubbery	42.818504	70.632586	27.05.1935	Chilikina L.	MW0813999
A. simile	Western Tien Shan, part of the Karatau Ridge Moist Depression	42.866667	70.600000	03.06.1936	Chilikina L.	MW0814000
A. simile	East Karatau	43.033333	70.350000	15.06.1936	Chilikina L.	MW0814001
A. simile	Tashkent district, Susak tract, foothills of the mountains 1600 m	41.233333	69.766667	31.05.1942	Whipper P.	MW0899995

A. simile	Western Tien Shan, spurs of the Chatkal range, Sumsar Gorge, rocky slopes	41.366667	71.116667	27.05.1958	Gubanov I.	MW0813995
A. simile	Chatkal ridge, southern slopes of the gorge. Tacha ata, Nanai, river valley	41.766667	71.633333	29.05.1958	Gubanov I. A.	MW0813993
A. simile	Kyrgyzstan, Chikan River basin, Kyzylkol river basin h-1900	42.133333	72.866667	10.07.1985	Klyuikov E. V.	MW0814006
A. simile	Kyrgyzstan, southern slope of the Kyrgyz Alatau range Kishi Kaindy River basin	42.664833	72.065392	11.06.1988	Klyuikov E. V.	MW0814004
A. simile	Jalabad region, northern slope of the Ferghana range Karasu River valley above the mouth of the Shaldyarak River	42.690019	72.062288	09.06.1996	Pimenov M. G.	MW0813990
A. simile	Kyrgyz Alatau ridge, northern gorges. Almalyk-sai gorge. 3rd gap to the west of the main gorge. Steppe slopes of the southern exposure, rocky places.	42.889052	71.727720	26.05.1961	Fisiun V. V.	AA
A. simile	Kyrgyz Alatau ridge, northern gorges. Almalyk-sai gorge. Settled meadows in the middle part of the ridge along the wide saddle of the Kainda mountain range.	42.883816	71.737177	01.06.1961	Gamayunova A. P.	AA
A. simile	Kyrgyz Alatau ridge, northern gorges. Almalyk-sai gorge, steppe slopes with shrubs, at the top of the spurs, height 1500 m.	42.882342	71.752820	23.05.1963	Gamayunova A. P.	AA
A. simile	North-western tip of the Kyrgyz Alatau range. Syugaty gorge in the upper reaches of the river. Up the steep northern slope.	42.873470	71.838125	07.06.1963	Goloskokov V. P.	AA
A. chychkanense	Kyrgyzstan, Koziyar River valley, spurs of the southern macroslope, Talas ridge, palogi slope, southern exposure height 2200 m.	42.351450	71.965908	28.07.1981	Ladygina G. M., Ikonnikov S. S.	LE
A. fetisowii	KZ. Almaty_Koktebe	43.541389	77.525024	16.05.2025	Friesen N, Abdildanov D.Sh.	AA
A. fetisowii	KZ. Almaty region, Karasai district	43.127278	76.512168	18.05.2025	Friesen N, Abdildanov D.Sh.	AA
A. fetisowii	KZ. Degeres	43.275617	75.781708	23.04.2024	Friesen N, Abdildanov D.Sh.	AA
A. simile	KZ. Kursai_KyrgyzAlatau	42.8624315	71.8461069	04.05.2025	Friesen N, Abdildanov D.Sh.	AA
A. simile	KZ. Makpal_KyrgyzAlatau	42.623830	72.890854	03.05.2025	Friesen N, Abdildanov D.Sh.	AA
A. simile	KZ. Usun Bulak_ KyrgyzAlatau	42.9135228	71.7615473	04.05.2025	Friesen N, Abdildanov D.Sh.	AA
A. simile	KZ. Sjugaty_KyrgyzAlatau	42.8624315	71.8461969	04.05.2025	Friesen N, Abdildanov D.Sh.	AA
A. simile	KZ. KaraArsha_KyrgyzAlatau	42.890988	71.824529	05.05.2025	Friesen N, Abdildanov D.Sh.	AA
A. simile	KZ. BolAlmalyKyrgyzAlatau	42.8862973	71.7286359	05.05.2025	Friesen N, Abdildanov D.Sh.	AA
A. simile	KZ. Tostanbek_KyrgyzAlatau	42.8378927	71.7171797	06.05.2025	Friesen N, Abdildanov D.Sh.	AA
A. simile	Kz. Kurdai	43.3370598	74.9284438	02.05.2025	Friesen N, Abdildanov D.Sh.	AA
A. simile	Kirgisischer Alatau ca. 20 km östlich Dzhambul, Nordhänge bei Uch-Bulak Tax 5052	42° 53	71° 35’	1998-05-12	Anonymous collectors	GAT13049

A.	*simile*	Paspel'dyk-Hügelmassiv SW Bishkek Tax 5685	42° 45‘	74° 33’	1998-05-12	Anonymous collectors	GAT13050
