# Supplementary figures and images for: Phylogeny and Taxonomy of Allium Section Longibidentata (R.M.Fritsch) R.M.Fritsch (A. Subgenus Melanocrommyum): Resurrection of Allium simile Regel
Source: Plants (Basel). 2026 Apr 22;15(9):1289. doi: 10.3390/plants15091289 (PMC13165040; doi:10.3390/plants15091289)

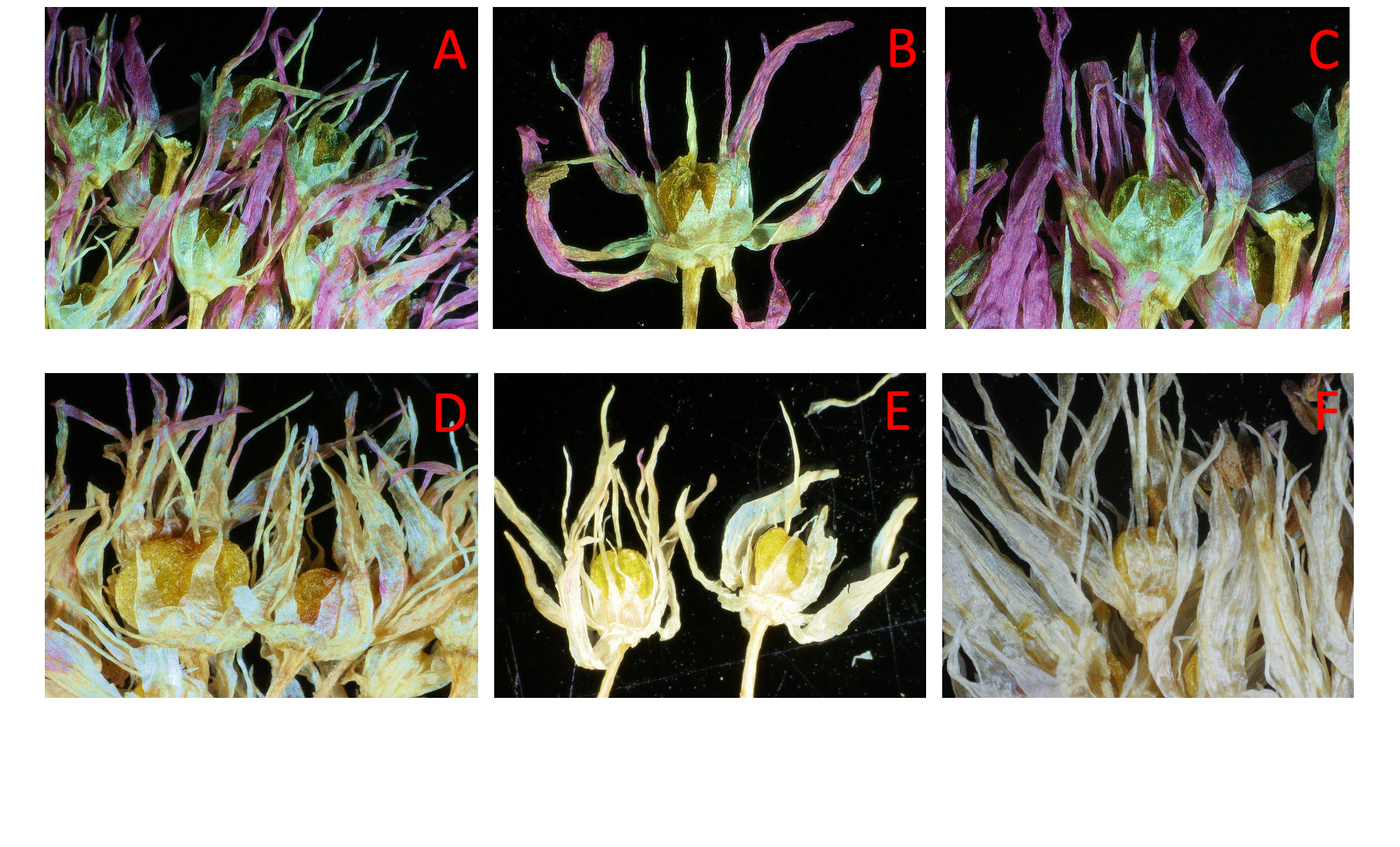

Supplement: Supplementary file 1 [file plants-15-01289-s001.zip › Supplementary S1.tiff]

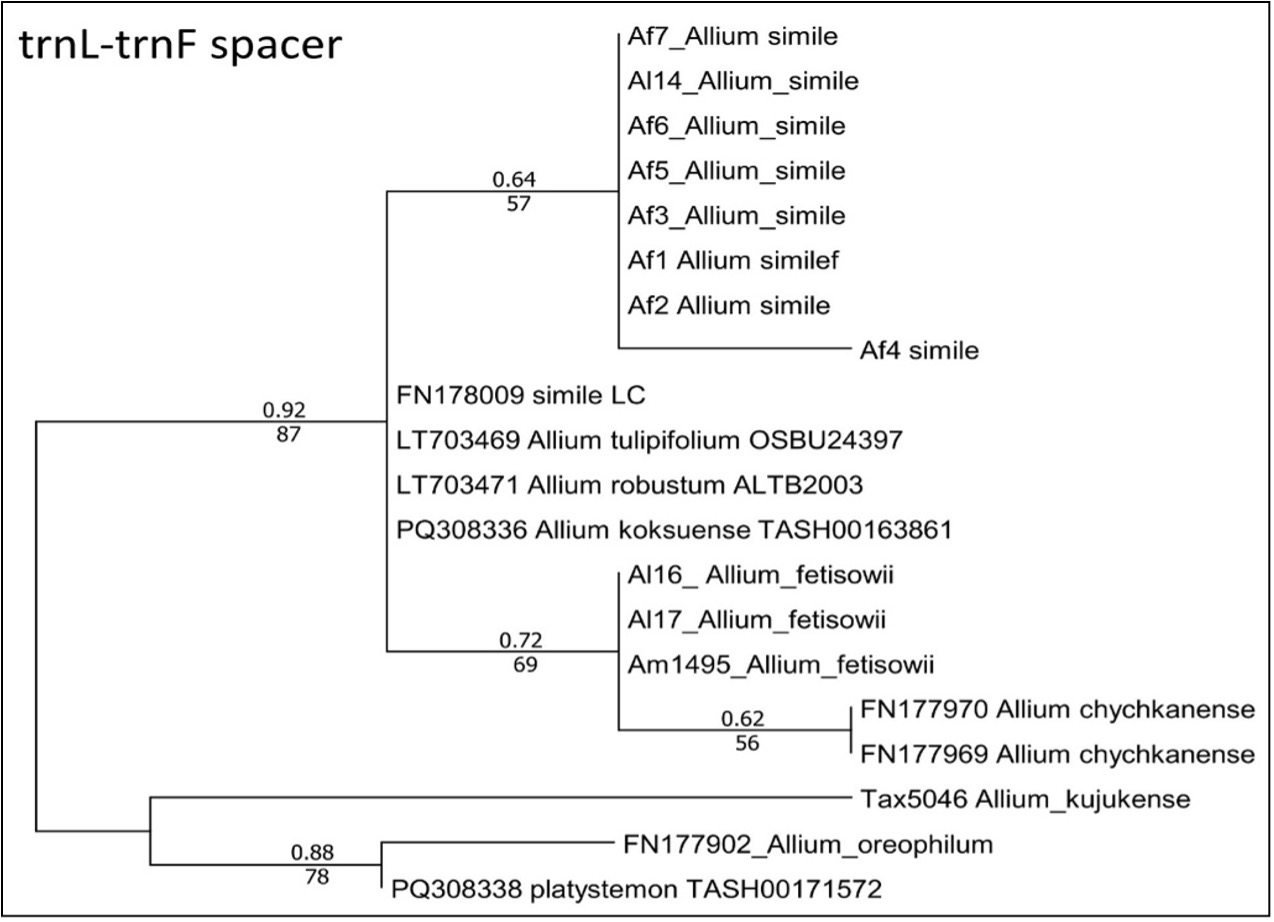

Supplement: Supplementary file 1 [file plants-15-01289-s001.zip › Supplementary S2-1.jpg]

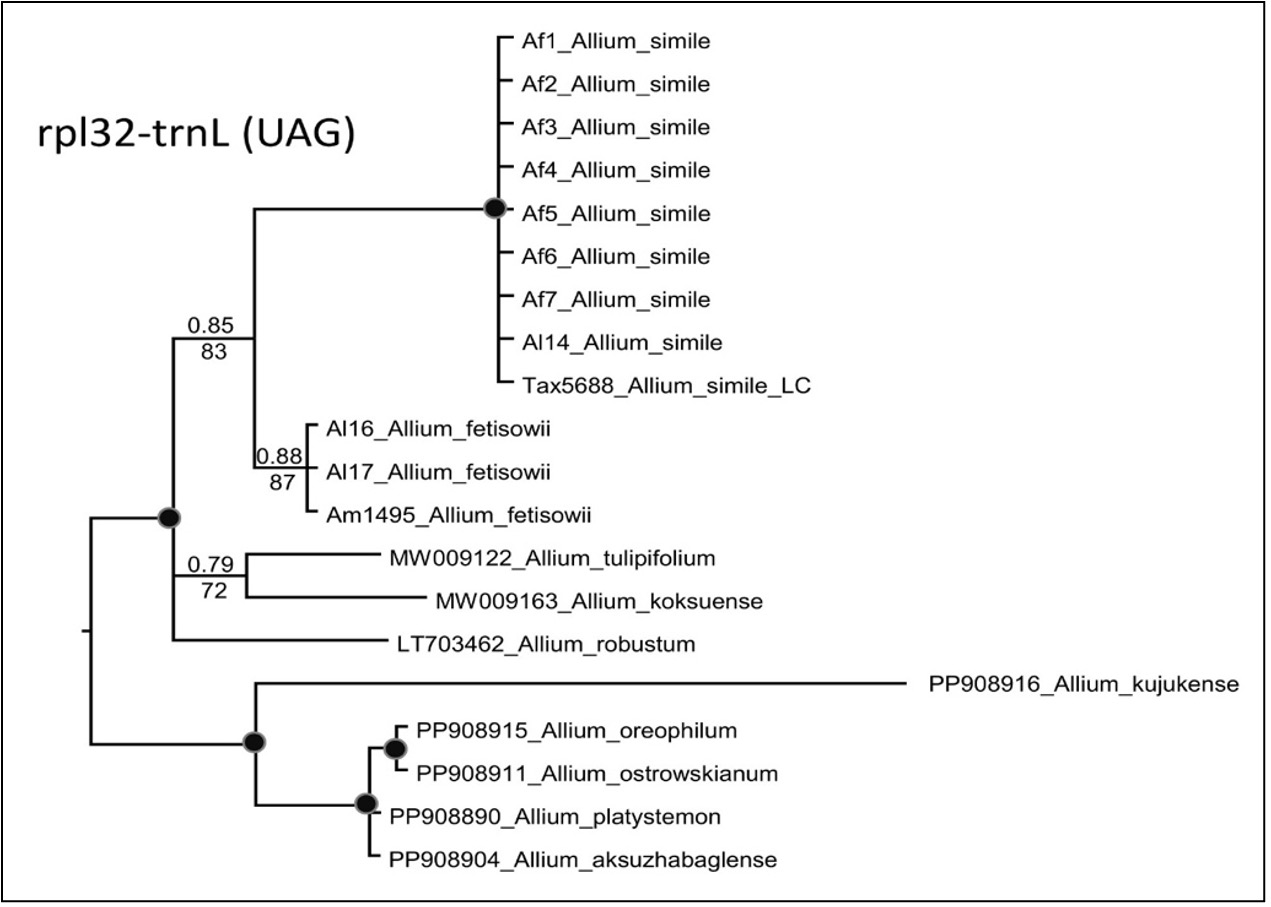

Supplement: Supplementary file 1 [file plants-15-01289-s001.zip › Supplementary S2-2.jpg]

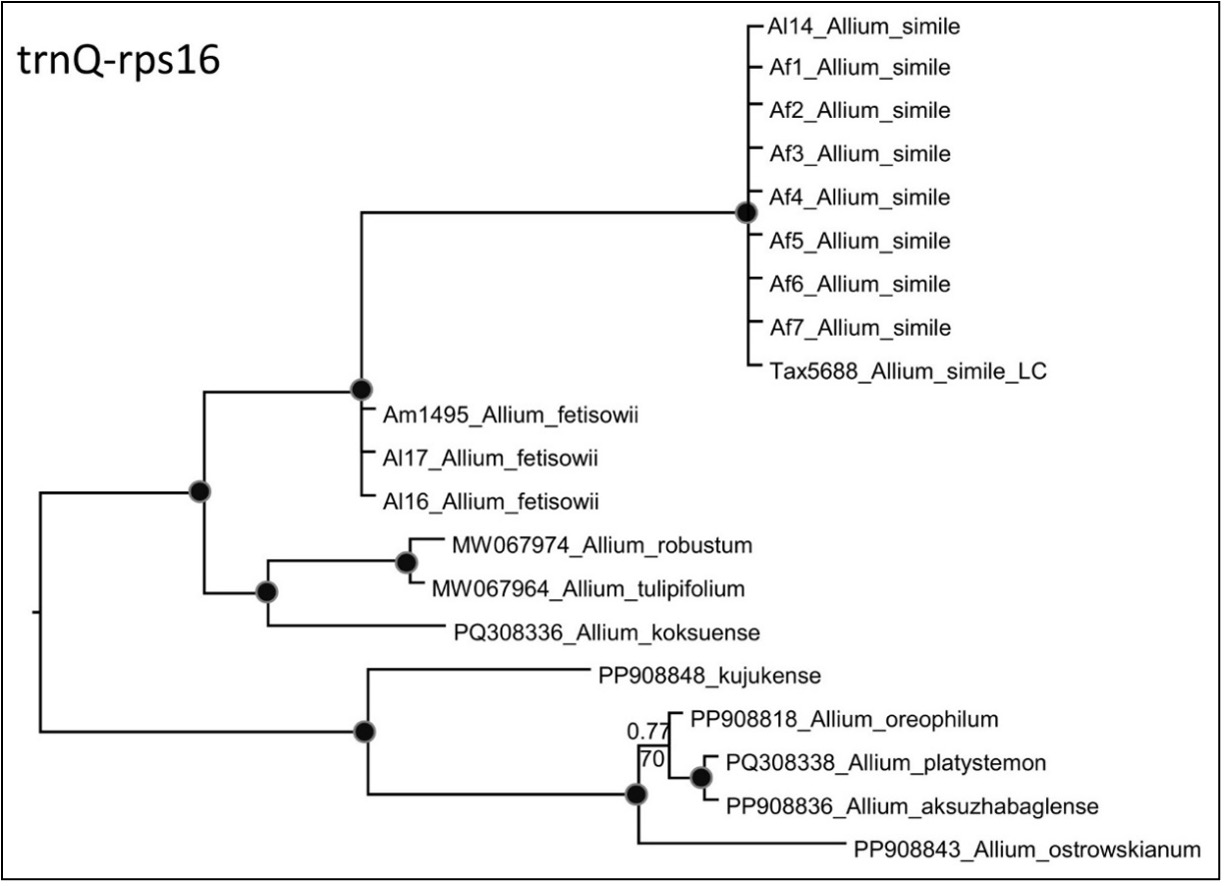

Supplement: Supplementary file 1 [file plants-15-01289-s001.zip › Supplementary S2-3.jpg]

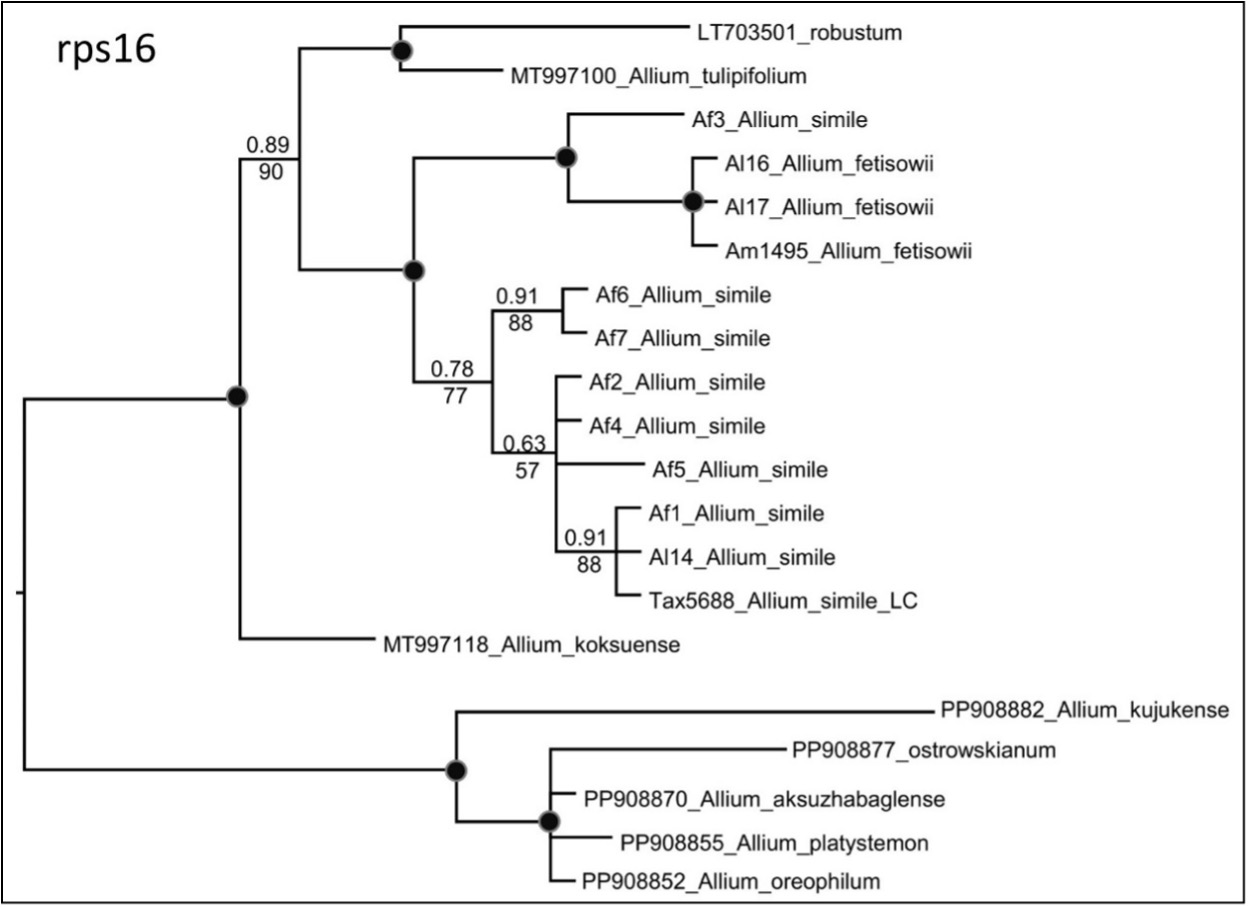

Supplement: Supplementary file 1 [file plants-15-01289-s001.zip › Supplementary S2-4.jpg]
